# Virtual Combinatorial Chemistry and Pharmacological Screening: A Short Guide to Drug Design

**DOI:** 10.3390/ijms23031620

**Published:** 2022-01-30

**Authors:** Beatriz Suay-García, Jose I. Bueso-Bordils, Antonio Falcó, Gerardo M. Antón-Fos, Pedro A. Alemán-López

**Affiliations:** 1 ESI International @ UCHCEU, Departamento de Matemáticas, Física y Ciencias Tecnológicas, Universidad Cardenal Herrera—CEU, CEU Universities San Bartolomé 55, Alfara del Patriarca, 46115 Valencia, Spain; afalco@uchceu.es; 2 Departamento de Farmacia, Universidad Cardenal Herrera—CEU, CEU Universities, C/Ramón y Cajal s/n, Alfara del Patriarca, 46115 Valencia, Spain; ganton@uchceu.es (G.M.A.-F.); paleman@uchceu.es (P.A.A.-L.); jose.bueso@uchceu.es (J.I.B.-B.)

**Keywords:** virtual combinatorial chemistry, virtual screening, QSAR, drug development

## Abstract

Traditionally, drug development involved the individual synthesis and biological evaluation of hundreds to thousands of compounds with the intention of highlighting their biological activity, selectivity, and bioavailability, as well as their low toxicity. On average, this process of new drug development involved, in addition to high economic costs, a period of several years before hopefully finding a drug with suitable characteristics to drive its commercialization. Therefore, the chemical synthesis of new compounds became the limiting step in the process of searching for or optimizing leads for new drug development. This need for large chemical libraries led to the birth of high-throughput synthesis methods and combinatorial chemistry. Virtual combinatorial chemistry is based on the same principle as real chemistry—many different compounds can be generated from a few building blocks at once. The difference lies in its speed, as millions of compounds can be produced in a few seconds. On the other hand, many virtual screening methods, such as QSAR (Quantitative Sturcture-Activity Relationship), pharmacophore models, and molecular docking, have been developed to study these libraries. These models allow for the selection of molecules to be synthesized and tested with a high probability of success. The virtual combinatorial chemistry–virtual screening tandem has become a fundamental tool in the process of searching for and developing a drug, as it allows the process to be accelerated with extraordinary economic savings.

## 1. Introduction

Traditionally, drug development included the individual synthesis and biological evaluation of hundreds of organic compounds with the intention of characterizing their biological activity, selectivity, bioavailability, and toxicity. On average, this process involved high economic costs and several years of research before identifying a drug with suitable characteristics to be commercialized [1]. Thus, the identification and synthesis of new compounds rapidly became the limiting step in the discovery and optimization of lead compounds for the development of new drugs [2]. In the past, chemical libraries used in biological assays were obtained by gathering compounds via purification and identification of biologically active ingredients from natural, marine, or fermentative products among other sources [3]. This was a time-consuming process that led to the appearance of combinatorial chemistry as a method to obtain large chemical libraries in a time-effective manner [2].

De Julian-Ortiz defined Virtual Combinatory Chemistry (VCC) as the computational simulation of the generation of new chemical structures by using a combinatorial strategy to generate a virtual library [4]. Since the generated compounds do not necessarily have to be new, VCC could be defined more precisely as computational simulation to generate structurally related compounds. Moreover, the concept of virtual combinatorial library should be clearly separated from databases in which compounds are not structurally related. In other words, a virtual combinatorial library can be generated by combining a limited number of chemical building blocks. The emergence of VCC, along with the publication of many databases with hundreds or thousands of compounds, has propelled the development of computational methods designed to analyze the rapidly increasing amounts of chemical information that is being generated [5]. Initially, these libraries or databases were analyzed using High-Throughput Screening (HTS), which involved the experimental screening of entire compound collections. However, the growing number of compounds available for screening promoted the development of computational approaches to complement HTS, such as Virtual Screening (VS) [6]. The main advantage of VS is that, while HTS requires experimentation to obtain results, VS consists in the computational evaluation of databases aiming to select a small number of reliable and experimentally testable candidate compounds that have a high probability of being active [5].

Different methodologies have been developed to carry out VS and they can be divided into two main categories: ligand-based VS (LBVS) and structure-based VS (SBVS) [7]. LBVS methods use the structural and biological data from a set of known active compounds to identify promising candidates for experimental screening [8]. These chemical data can be based on either 2D or 3D representations of the molecules. On the other hand, SBVS requires the 3D representation of the target, as this approach aims to find molecules that fit within a binding site in the best position and orientation possible [9].

Furthermore, besides identifying the appropriate chemical structure, other factors must be considered during the drug design process. For example, variations in crystal structure can lead to different polymorphs of a solid compound with different physicochemical characteristics that can translate to pharmacokinetic differences that, in turn, may affect their activity [10,11,12]. For this reason, understanding crystallization has become increasingly important to have a reproducible drug production process. In fact, Density Functional Theory (DFT) has become increasingly popular in drug design because it can predict this behavior in active pharmaceutical ingredients, among many other things [13].

This review discusses chemical combinatorial libraries as well as other existing databases available for VS and the different methodologies used for VS. This review is divided into three main parts. In the first part, we analyze the different strategies used to generate virtual combinatorial libraries as well as the methods that can be used to do so. In the second part, we review the methodologies used to carry out the virtual screening of combinatorial libraries and non-combinatorial databases. Lastly, the third part includes examples and applications of the aforementioned methodologies in the discovery and development of new drugs.

## 2. Virtual Combinatorial Library Creation

The design of virtual combinatorial libraries (VCLs) is a critical part in the early phases of the drug discovery process as these libraries are used in lead generation projects to identify series of analogues around hit and lead compounds to explore structure–activity relationships (SARs) [14]. Starting from a single known bioactive molecule acting as a template, a set of theoretically isofunctional molecules can be virtually assembled mimicking the pharmacophore pattern [15]. In the following, we discuss the different approaches that can be followed to create a VCL as well as the different software platforms available to do so.

### 2.1. Types of Combinatorial Libraries

There are two main classifications of VCLs regarding their generation process: based on a synthetic route or based on a scaffold structure.

The synthetic route approach starts with the identification of the chemical reactions intended to be followed to obtain the designed compounds. This includes the reaction rules, the reaction strategy, allowed products, forbidden products, parameter values that define the logical conditions for reaction application, and the sites where reactions occur [16]. Basically, the library is made up of the products of carrying out a certain reaction with *n* reactants of type A and *n* reactants of type B. This approach imitates quite accurately the steps followed in real chemical synthesis. In fact, the similarity it has with in situ chemical synthesis is the reason why this is the approach generally followed by the pharmaceutical industry. Examples of the application of reaction-based VCLs in the pharmaceutical industry include BI-Claim developed by Boehringer Ingelheim, Eli Lilly’s Proximal Collection, and Pfizer global virtual library (PGVL) [17,18,19]. All these VCLs were built using prevalidated or reported reactions as well as accessible chemical reagents. Similarly, Humbeck et al. developed CHIPMUNCK, a VCL that covers over 95 million compounds [20]. This combinatorial database is composed of three sub-libraries, each being the product of a special set of in-silico-performed reactions: heterocycle forming reactions, medicinal chemistry reactions, and multicomponent reactions. Another example of a VCL based on a synthetic route is ZINClick [21]. This combinatorial library contains over 16 million 1,4-disubstituted-1,2,3-triazoles that can by synthesized via a “click” 1,3-dipolar cycloaddition reaction between azides and alkynes catalyzed by copper salts. Similarly, Saldívar-González et al. applied a Diversity-Oriented Synthesis strategy to design a library of lactams that could be easily synthesized by performing a series of intramolecular paring reactions to form an amide bond between carboxylic acids and primary or secondary amines [22].

The other main approach to VCL design is that based on a scaffold structure. This method consists in the determination of a common skeleton with variable sites tagged as R_1_, R_2_, R_3_… R_n_, where each one is associated with a list of possible substituents [23]. This approach is ideal in those cases where there are different synthetic routes described to obtain a common scaffold [24]. This type of VCL is focused on a specific target, structural class, or pharmacophore as it stresses the exploration of a specific area of the chemical space, resulting in a small number of structurally related compounds based on a known target or family [24]. Examples of this type of VCL include the combinatorial library of 1001 6-fluoroquinolones developed by Bueso-Bordils et al. [25] to identify new compounds with antibacterial activity against methicillin-resistant *Staphylococcus aureus* (MRSA). The library was built using a 6-fluoroquinolone skeleton with structural variations in positions 1, 7, and 8. Similarly, Kouman et al. designed a VCL based on a benzamide scaffold to identify new *Mycobacterium tuberculosis* 2-trans enoyl-acyl carrier protein reductase inhibitors with favorable pharmacokinetic profiles [26]. Lauro et al. have also built a library containing approximately 2.0 × 10^4^ virtual compounds by following a multicomponent-based chemical route for the decoration of the 2,4-thiazolidinedione core [27].

### 2.2. Generation of Combinatorial Libraries

Virtual combinatorial libraries can be generated using different computational tools and software [28]. Table 1 summarizes different tools that can be used to build VCLs of small molecules. Some of these tools, such as KNIME, RDKit, DataWarrior, and Reactor, allow for the creation of a VCL based on a list of prevalidated reactions [29,30,31,32,33,34]. Others, such as Library Synthesizer, SimLib, MOE, Schrödinger, and Nova, use the scaffold-based approach to create the combinatorial library by allowing the user to select a common scaffold or molecular skeleton with tagged substitution points to which different R groups will be attached [35,36,37,38,39,40]. Finally, a third type of model includes those using multi-objective algorithms such as CCLab and MoSELECT [29,30]. In this case, the tool does not only provide a set of combinatorial compounds, but also provides filtering options regarding aspects such as synthesis cost, drug-likeness, physicochemical properties, and structural diversity. These tools allow the relationship between different objectives to be explored with competing objectives easily identified. Thus, the library designer can make an informed choice on which solution to explore.

## 3. Virtual Screening

Virtual screening can be defined as a computational technique that is generally used in the early stages of the drug discovery process to search libraries of small molecules to identify chemical compounds that are likely to bind to one or several drug targets [42]. In other words, VS is a step-by-step method with a series of filters able to narrow down and choose a set of lead-like hits with potential biological activity against intended drug targets [43]. Essentially, VS could be considered as an experimental high-throughput screening (HTS) performed in silico [44]. VS presents two main advantages when compared to the traditional experimental HTS. Firstly, it acts as a filter, selecting only those candidates with the most favorable characteristics to be active, which can then be tested in vitro. This leads to the second main advantage, which is the fact that, since the compounds studied do not necessarily exist, their “testing” does not consume valuable substance material, which, in turn, improves the time- and cost-effectiveness of the drug development process. Therefore, any molecule can, in theory, be evaluated using VS.

### 3.1. Methods Used in Virtual Screening

Virtual screening techniques can be grouped into two major categories, depending on the type of information used to develop the screening models. Ligand-based virtual screening relies on structural and physicochemical properties of the chemical scaffold of known active and inactive molecules and is based on the molecular similarity principle [7]. On the other hand, SBVS exploits the three-dimensional structure of the target protein [9]. In the following, we will describe different methodologies used in LBVS and SBVS.

#### 3.1.1. Ligand-Based Virtual Screening (LBVS)

As was mentioned above, LBVS is based on molecular similarity through the comparison of different structural and physicochemical properties [7]. The main hypothesis behind LBVS is that similar compounds will cause similar biological effects. Essentially, large ligand libraries are searched to identify compounds with similar chemical properties or shapes to molecules with known pharmacological activity, which can in turn result in the identification of new active compounds [45]. The search can be performed using several screening methods that differ on the measure of similarity, ranging from two-dimensional descriptors to shape comparisons and three-dimensional descriptors.

Quantitative Structure–Activity Relationship (QSAR) models are one of the main methods used in LBVS. These models can identify the correlation between structure-based molecular descriptors and biological activity [46]. Traditionally, these models were used retrospectively, with scientists focused on developing explanatory models of existing data [47]. However, the substantial increase in the size of experimental datasets available has led to an increase in the use of QSAR models as a virtual screening tool to discover active compounds in chemical databases and VCLs [48]. There are many QSAR approaches that differ on the structural parameters, also known as descriptors, used to characterize molecules as well as on the mathematical approaches used to establish the correlation between descriptor values and pharmacological activity [49].

The molecular descriptors used in QSAR models can be divided into five groups: topological, geometrical, thermodynamic, electronic, and constitutional [50,51,52]. Topological and geometrical descriptors represent the connectivity of atoms in a molecule as well as its shape but, while topological descriptors are based on 2D molecular graphs, geometrical descriptors are calculated from the 3D coordinates of the atoms. Thermodynamic descriptors relate the chemical structure to an observed chemical behavior. Examples of these include molar refractivity as a combined measure of molecular size and polarizability, log *P* to characterize the hydrophobicity of the molecule, and solvation free energies [53]. Electronic descriptors describe electronic aspects of the molecule or atom bonds such as the charge distribution in a molecule. Lastly, constitutional descriptors reflect simple chemical information about a molecule, such as the molecular weight or the number of bonds in the molecule.

There are many mathematical methods used to build the QSAR predictive models. These could be grouped into linear and machine learning approaches [54]. Linear methods, which include linear discriminant analysis, multiple linear regression, and partial least squares, among others, fit data to an equation and report the coefficients derived from it. On the other hand, machine learning methods, among which one can find neural networks and support vector machines, process input information and recognize patterns.

Another widely used LBVS approach is pharmacophore-based modeling. In this case, different algorithms are applied to identify configurations or spatial arrangements of chemical features that are common to molecules with a known activity [55]. These chemical features include, but are not limited to, hydrogen bonds, charges, and hydrophobic areas [56]. The analysis can be carried out in either a 2D or 3D space [57]. Pharmacophore models are based on the principle that novel compounds able to fulfill a certain interaction pattern regarding the aforementioned chemical features should bind and show comparable biological activity to that of the known active molecule. Pharmacophore modeling starts with the identification of the pharmacophore of a molecule with a desired activity. Subsequently, a conformational analysis is carried out where the flexibility of small molecules is handled by enumerating multiple conformations for each molecule in the database. Pharmacophore-based LBVS can sometimes be confused with molecular docking, an SBVS method. The main differences between them will be discussed after molecular docking is explained.

#### 3.1.2. Structure-Based Virtual Screening (SBVS)

SBVS, also known as target-based virtual screening (TBVS), aims to predict the best interaction between ligands and a molecular target to form a complex [9]. In other words, the affinity of different ligands to the target is assessed and ranked. Thus, to perform SBVS, the 3D structure of the target protein must be known to be able to predict the interactions between the target and each chemical compound in silico [58]. This technique is based on a series of algorithms that explore the geometrically feasible alignments of different ligands with a specific drug target [59]. As a result, the ligands are ranked according to their affinity with the receptor site, allowing for the identification of molecules that are more likely to present pharmacological activity. In order to carry out this ranking, scoring functions are used to approximate the binding free energy between the protein and the ligand in each docking pose [60]. Lastly, the results are processed to examine the validity of the generated pose, undesirable chemical moieties, metabolic liabilities, desired physicochemical properties, lead-likeness, and chemical diversity [61].

Scoring functions play a key role in molecular docking. These functions can be divided into three categories: empirical, knowledge-based, and physics-based [62]. Empirical functions are some of the most widely used as they are easy to compute. These functions try to capture relevant elements of binding free energy, such as solvent accessible surface, entropy, and hydrogen bonds, to then fit them in experimental data [63]. In fact, because of their simple energy terms, these scoring functions are able to predict binding affinity, ligand pose, and virtual screening with low computing costs; however, their accuracy is lower compared with the other two types of functions [64]. On the other hand, knowledge-based scoring functions calculate the desired pairwise potentials from three-dimensional structures of a large set of protein–ligand complexes based on the inverse Boltzmann statistic principle [65]. In this case, the size and quality of the databases used to derive the statistical potentials have a great impact on the accuracy of knowledge-based scoring functions. Lastly, physics-based scoring functions include scoring functions based on force field, solvation model, and quantum mechanics methods [66,67,68]. These scoring functions can directly compute the interactions between the atoms of a protein and ligand, having a greater predictive accuracy than other types of scoring functions due to consideration of the enthalpy, solvation, and entropy.

Having seen molecular docking and pharmacophore-based VS, it is easy to confuse one with the other as both aim to identify molecules capable of binding to a certain drug target. However, their difference relies, essentially, on the methodology. While pharmacophore-based VS uses the structures of ligands with known pharmacological activity to predict chemical structures that should bind to proteins in the same way, molecular docking requires the defined 3D structure of the target protein to study which compounds will bind more effectively to it and, thus, have the higher probability of being pharmacologically active [64,69].

## 4. Applications and Current Trends

**Figure 1:**
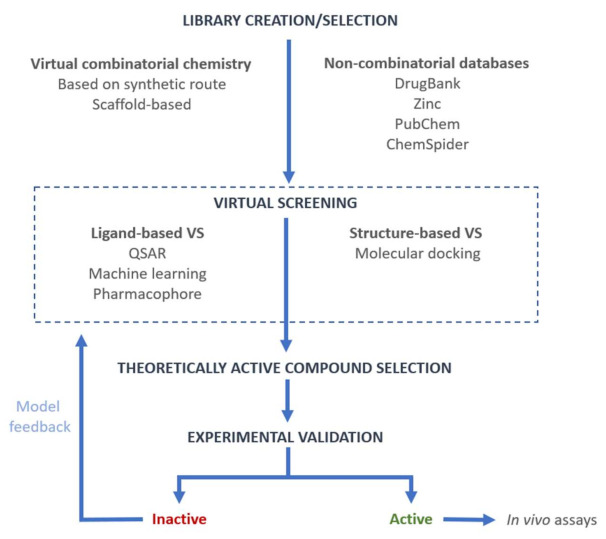
General flowchart used in virtual screening.

The different methodologies of VS have been widely used for the discovery and development of new drugs. This VS can be either performed on virtual combinatorial libraries or on large databases of chemical compounds available online (Figure 1). The number of chemical databases available for VS has increased exponentially in the last few years as the advances in computational methods have vastly increased the information output [70]. These databases include chemical, biomolecular, drug–target interaction, and/or disease information and can be used for drug discovery and drug repurposing. Some of the most widely used databases in medicinal chemistry include PubChem, ZINC, ChemSpider, and DrugBank [71,72,73,74]. In the following, we will present successful examples of the different VS techniques applied in both VCLs and chemical databases for the discovery of new drugs in the early stages of the development process.

As was mentioned earlier, QSAR models were initially used to interpret the structure–activity relationship of lead compounds. However, this technique evolved and QSAR models began to be applied in the prediction of pharmacological activity. For example, Bueso-Bordils et al. built a QSAR model based on linear discriminant analysis to predict antibacterial activity against MRSA [25]. They used this model to virtually screen a fluoroquinolone VCL, identifying 117 theoretically active molecules of which five were synthesized and three showed anti-MRSA activity comparable to that of ciprofloxacin. Similarly, Suay-Garcia et al. developed a tree-based QSAR model based on quinolones that was applied to the DrugBank database to screen for active compounds against *Escherichia coli* [75]. The model identified 134 drugs with theoretical activity against *E. coli* of which eight were already commercialized as antibacterial drugs, 67 were approved for different pathologies, and 55 were drugs in experimental stages. The same methodology was used by Luo et al. to develop a binary classification QSAR prediction model that was used to mine drug-like, diversity, and GPCR-targeted libraries to identify novel anxiolytics and potential antischizophrenic drugs [76]. Another QSAR model was developed using GUSAR software to identify novel HIV-1 integrase inhibitors [77]. This model was used to virtually screen a subset of 308 structurally distinct compounds from the BindingDB database. Of these, 236 compounds were selected as potential candidates for synthesis due to their good druglikeness. Finally, six compounds were chosen to be synthesized and one of them was experimentally confirmed to inhibit the strand transfer reaction in HIV. More recently, Zaki et al. developed a balanced QSAR model based on the genetic similarity between SARS-CoV-2 and SARS-CoV to identify novel molecules with inhibitory potential against the main protease of SARS-CoV-2 [78]. The study combines a prediction QSAR model along with molecular docking and molecular dynamics to screen 26,467 food compounds and 360 heterocyclic variants of a benzotriazole–indole hybrid molecule to identify promising hits to treat COVID-19.

Pharmcophore-based models are the other most common LBVS approach in virtual screening. For instance, a pharmacophore-based model was developed to identify potential σ1 receptor ligands to treat Alzheimer’s Disease [79]. This model was applied to screen 8543 compounds from the Life Chemicals database, of which five candidates presented excellent druglikeness and ADMET properties. Along these lines, Liu et al. generated a pharmacophore model from the structures of active amino alcohols to perform a virtual screening to discover novel compounds with anti-echinococcal activity [80]. The screening was performed on the ZINC15 database and, out of the 62 compounds selected by the model, 10 were found to be experimentally active against *Echinococcus multilocularis*. Kouman et al. followed a similar procedure to identify benzamides capable of inhibiting 2-trans enoyl-acyl carrier protein reductases in *Mycobacterium tuberculosis* [26]. In this case, a pharmacophore model generated from the active conformations of N-benzyl-4-((heteroaryl)methyl) benzamides (BHMBs) was used as a virtual screening tool of novel analogs included in a VCL of compounds containing benzamide scaffolds. The model identified 90 new and potent BHMBs with enhanced cell membrane permeability and high human oral absorption compared with current treatments for tuberculosis. Screening of a virtual combinatorial library with a pharmacophore model was also used to identify novel µ-opioid receptor inverse agonists to treat narcotic overdose or drug addiction [81]. More specifically, a library including 19,800 tetrapeptides was created to perform the virtual screening and three candidates were selected for binding assays. 

Regarding SBVS, molecular docking is the most widely used technique. However, the latest VS trends aim for a consensus approach in which different VS techniques are used in combination to optimize results. Thus, molecular docking is generally found to be used along with LBVS models. For example, a combination of a pharmacophore-based model with 3D-QSAR and molecular docking was used to virtually screen the ZINC and ASINEX databases to identify potential dipeptidyl peptidase IV inhibitors to be used as oral antidiabetics [82]. More specifically, the pharmacophore and 3D QSAR model was used to virtually screen the aforementioned databases and the hit molecules were used to design a VCL that was evaluated using molecular docking. A similar procedure was followed by Bommu et al. to predict potential epigallocatechin gallate (EGCG) analogs against epidermal growth factor receptors [83]. In this case, log *P* and log *S* predictions along with the toxicity endpoint were modeled using QSAR, which was combined with a pharmacophore model and molecular docking to identify seven high-potential EGCG analogs as promising pharmacological, anticancer, and drug-like templates that could be used towards moderating lung cancer progression. This consensus approach was also used to identify natural compounds against mosquito-borne Chikungunya virus targets [84]. To do so, a subset of compounds from natural sources found on PubChem was studied using molecular docking and the selected potential ligands were subjected to 3D-QSAR studies to predict biological activity. Finally, Lipinski’s rule and ADMET studies were also performed, leading to the identification of the four best-fit compounds of natural origin against targets of the Chikungunya virus.

## 5. Conclusions

Virtual Combinatorial Chemistry and the different Virtual Screening tools are presented as a key tool in the development of new drugs in a time- and cost-effective manner. These in silico methods, whether combined or on their own, accelerate the drug discovery process by acting as filters and allowing experimental evaluation to be focused only on compounds with the most drug-likeness.

## Figures and Tables

**Table 1:** Examples of chemoinformatic tools available to create chemical libraries of small molecules. (Adapted from Saldívar-González et al. [28]).

Tool/Software	Main Features	Ref.
CCLab	Based on a multi-objective genetic algorithm, including synthesis cost and drug-likeness.	[29]
MoSELECT	Based on a multi-objective genetic algorithm, including diversity and “drug-like” physicochemical properties, and a fitness function.	[30]
KNIME	Based on generic reactions.	[31]
RDKit	Based on generic reactions.	[32]
DataWarrior	Molecules are designed following a given generic reaction and a list of real reactant structures.	[33]
Library synthesizer	Creates libraries through specification of a central scaffold with connection points and a list of R groups.	[35]
SimLib v2.0	Libraries are built using SMILES and a scaffold-based approach.	[36]
GLARE	Allows one to optimize reagent lists for the design of combinatorial libraries.	[41]
Reactor (ChemAxon)	Library generated using generic reactions and considering reaction rules that yield chemically feasible products.	[34]
Molecular Operating Environment (MOE)	Scaffold-based. New chemical compounds are generated by attaching R groups to a common skeleton with marked points.	[37]
Schrödinger	Creates library by substituting attachments on a core structure with fragments from reagent compounds.	[38]
Nova	Uses central scaffolds and a list of R groups.	[39]
ChemDraw	Uses central scaffolds and a list of R groups.	[40]
